# Are rural adolescent girls in Senegal more likely to use family planning? DHS 2023 analysis

**DOI:** 10.4102/jphia.v16i1.1345

**Published:** 2025-10-23

**Authors:** Ndeye M. Sougou, Cheikh T. Diop

**Affiliations:** 1 Department of Preventive Medicine and Public Health, Faculty of Medicine, University Cheikh Anta Diop, Dakar, Senegal; 2 Institute of Health Development, Faculty of Medicine, University of Cheikh Anta Diop, Dakar, Senegal; 3 International Research Laboratory (IRL 3189 ‘Environnement, Santé, Sociétés’), Faculty of Medicine, University of Cheikh Anta Diop, Dakar, Senegal; 4 Department of Public Health, Faculty of Medicine, University Alioune Diop of Bambey, Bambey, Senegal

**Keywords:** family planning, adolescent girls, associated factors, adolescents’ sources of family planning information, Senegal

## Abstract

**Background:**

In Senegal, adolescent reproductive health is a public health priority.

**Aim:**

The objective of this research is to identify and analyse the key determinants of modern contraceptive uptake among Senegalese adolescents in 2023.

**Setting:**

The study focused on adolescent family planning in Senegal in 2023.

**Methods:**

The study draws on secondary data from the 2023 Senegal Demographic and Health Survey. The sample comprised 16 583 women aged 15–49 years. Adolescent girls aged 15–19 years represented 4024 individuals. The analysis included descriptive, comparative and multivariable statistical approaches. The outcome variable was binary, indicating whether or not a modern contraceptive method was used. All analyses were conducted using STATA version 15.

**Results:**

Among the adolescent girls surveyed, 19.33% reported having initiated sexual intercourse, with the earliest reported age at sexual debut being 9 years. Additionally, 10.77% had engaged in sexual activity within the 4 weeks preceding the survey, indicating recent sexual activity. However, only 2.05% (*n* = 80) of these adolescents reported using contraception at the time of the survey. Factors significantly associated with the use of modern contraceptive methods included residing in a rural area (adjusted odds ratio [AOR] = 0.58; 95% confidence interval [CI]: 0.34–0.96), being married (AOR = 20.61; 95% CI: 11.43–37.14), low socioeconomic status (AOR = 2.35; 95% CI: 1.35–4.08), and identifying as Christian (AOR = 3.41; 95% CI: 2.52–18.19).

**Conclusion:**

These findings underscore the need for targeted and context-sensitive sexual and reproductive health interventions. In particular, efforts should prioritise improving access to modern contraception among the most vulnerable subgroups, including adolescent girls living in rural areas, those from socio-economically disadvantaged backgrounds, and unmarried adolescents.

**Contribution:**

This study provides a better understanding of the determinants of family planning among adolescents, which can inform the development of evidence-based health programmes aimed at improving the sexual and reproductive health of Senegalese women.

## Introduction

Family planning is a proven public health intervention that contributes significantly to reducing maternal and child mortality and enhancing the overall quality of life. Ensuring access to modern contraception for women and girls is not only vital for the health and well-being of individuals but also instrumental in achieving sustainable development, unlocking the economic potential of nations and safeguarding the health of future generations.^1^ Modern contraceptive methods constitute a cornerstone of both primary and reproductive health care. They are essential for enhancing health outcomes among mothers, newborns and children by decreasing disease burden and death rates within these populations, and by helping to limit the spread of human immunodeficiency virus (HIV) and/or acquired immunodeficiency syndrome (AIDS).^2^ Among available interventions, contraception is the principal tool enabling women and girls to exercise control over their reproductive lives, including the timing and spacing of pregnancies.

As of recent estimates, approximately 705 million women in low- and lower-middle-income countries are using contraceptive methods, thereby preventing an estimated 376m unintended pregnancies each year. These preventive measures, in turn, help avert approximately 39m miscarriages, 2m stillbirths, and 131 000 maternal deaths each year.^3^

In Senegal, the legal and policy framework surrounding contraception has evolved significantly over the past few decades. The 1980 abolition of the 1920 law prohibiting the promotion and use of contraception represented a turning point in reproductive health policy,^4^ followed by the integration of family planning services into maternal and child health programmes. In 1988, the government adopted a national population policy with the explicit goals of reducing fertility rates and population growth. Key strategies included the promotion of contraceptive use, the dissemination of family planning methods, and the introduction of population education programmes to prevent adolescent pregnancies.^5^

Despite these efforts, the uptake of modern contraception has shown signs of stagnation in recent years. After rising to 26.3% in 2017, the modern contraceptive prevalence rate (mCPR) fell to 25.4% in 2018, following a plateau in 2016 at 23.7%.^6^ Several studies have identified persistent barriers to contraceptive use, notably sociocultural constraints and weaknesses in the health system.^7,8,9^ However, these studies often overlook other key determinants of health that may influence contraceptive adoption, particularly among adolescents, such as limited knowledge, decision-making autonomy, peer and partner influence, perceived quality of services, and financial accessibility.

Adolescent girls remain a vulnerable population with substantial unmet needs in sexual and reproductive health.^10^ They face numerous obstacles in accessing contraceptive services, including social stigma, limited autonomy, and inadequate youth-friendly services.^11^ While early sexual debut and child marriage remain prevalent among adolescent girls in Senegal, as in many sub-Saharan African countries,^12^ the literature remains limited regarding their specific access to contraception.

It is within this context that the present study aims to examine the determinants of modern contraceptive use among adolescent girls in Senegal in 2023. The findings are expected to inform the development of targeted strategies and interventions tailored to this particularly vulnerable population group.

## Research methods and design

### Type of study

This was a descriptive cross-sectional study using data from the 2023 Demographic Health Survey (DHS).

### Study population

These were teenage girls aged 15–19 years, living in Senegal in 2023.

### Inclusion criteria

Teenage girls aged between 15 years and 19 years, residing in Senegal, who were present in the household on the night before the survey.

### Eligibility limitations

Individuals who refused to be interviewed were not considered eligible for inclusion.

### Survey design

Following DHS standards, a two-stage stratified sampling approach was used to ensure national, regional, and urban and rural representativeness across all 14 regions of Senegal. Sampling was implemented separately within each stratum. During the initial phase of the Senegal DHS sampling process, 400 primary sampling units (PSUs) were systematically selected from Enumeration Zones defined by the Recensement Général de la Population, de l’Habitat, de l’Agriculture et de l’Élevage (RGPHAE) census, using a probability proportional to size method based on the number of households per zone. A count of households in each of these clusters provided a list of households from which a sample of 22 households per cluster was drawn in the second stage, in both urban and rural areas, using a systematic draw with equal probability.

The analysis was based on the Individual Recode dataset, which includes women aged 15–49 years who were present in the household on the night preceding the survey interview.

### Variables

The target population for this study consisted of female adolescents aged 15–19 years. The outcome variable analysed was the use of a modern contraceptive method, recorded in the DHS as a binary variable (yes or no).

Several explanatory variables were considered, encompassing individual characteristics, sociodemographic attributes, and economic conditions.

#### Individual factors

These included the teenager’s marital status (divorced, married, widowed) and her religion.

#### Sociodemographic and economic factors

The mother’s educational attainment was categorised into four levels: no formal education (referring to those who never attended the French formal school system), primary, secondary, and higher education. The wealth index, a measure of relative economic well-being based on household assets, was classified into quintiles (lowest, second, middle, fourth, highest) and derived from the wealth score. Place of residence (urban versus rural) was also taken into account.

### Analysis

Data analysis was performed using STATA/SE version 15.1. As previously described in the data sources section, the study employed a two-stage sampling strategy. To address the complexity of the sampling design, weighting adjustments were applied to correct for unequal selection probabilities and non-response bias.

In the descriptive phase, data were summarised using proportions and absolute counts. The Chi-square test (χ^2^) was employed to evaluate associations between categorical variables. A *p*-value below 0.05 was considered statistically significant, with 95% confidence intervals [CI] used for precision estimates. Variables with a *p*-value less than 0.25 in the bivariate analysis were included in the multivariate model, in line with the recommendations of Hosmer and Lemeshow, to avoid excluding potentially important variables that may become significant after adjustment for confounder.^13^

In order to identify factors linked to modern contraceptive use among adolescents, a multivariable logistic regression was conducted, adjusting for potential confounders. Adjusted odds ratios (s) were estimated along with their 95% CIs. Given the survey’s complex design – incorporating stratification, weighting, and clustering – the analysis specified design variables for weights, strata, and PSUs using the svy prefix in STATA.

**FIGURE 1:**
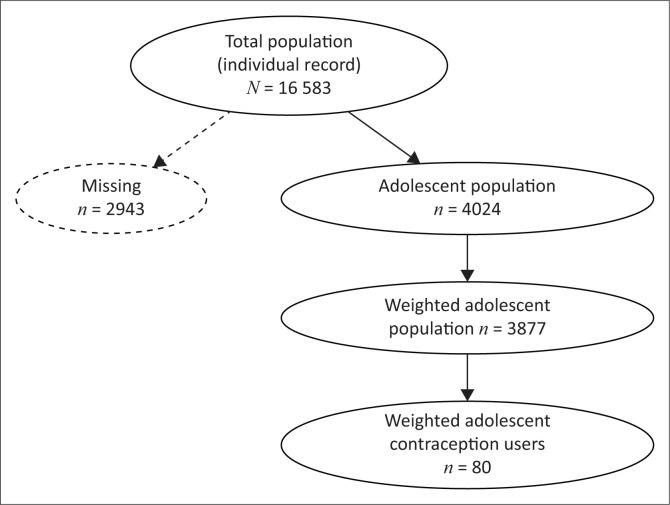
Diagram of study flow.

The survey also identified 16 583 women aged 15–49 years. Teenage girls aged 15–19 years accounted for 4024 individuals. After allocation of the survey weight, they numbered 3077 (see Figure 1 [data flow diagram]).

### Ethical considerations

Ethical clearance to conduct this study was obtained from the ICF Macro Institutional Review Board (No. 31561.00.000.00).

## Results

The results presented below provide an overview of the sociodemographic characteristics of the study population and describe patterns of sexual activity and modern contraceptive use among adolescent girls in Senegal. This section also highlights the key factors statistically associated with contraceptive use, based on both descriptive and multivariate analyses.

### Sexual behaviour and practices

Adolescent girls who had sexual intercourse accounted for 19.33% (see Table 1). The youngest age recorded for sexual initiation was 9 years (Table 1). A total of 10.77% of adolescent girls had been sexually active in the past 4 weeks (see Table 2).

**TABLE 1:** Age at 1st sexual intercourse.

Age at 1st sexual intercourse (years)	*n*	%
No sexual intercourse	3122	80.54
9	1	0.03
10	3	0.09
11	12	0.30
12	13	0.34
13	41	1.06
14	92	2.37
15	161	4.15
16	154	3.97
17	150	3.88
18	97	2.51
19	25	0.63

**TABLE 2:** Recent sexual activity in adolescents (*N* = 3877).

Recent sexual activity	*n*	%
Never had sex	3122	80.54
Active in the last 4 weeks	417	10.77
Not active in the last 4 weeks – postpartum	107	2.76
Not active in the last 4 weeks – not postpartum	230	5.94

### Method of contraception

Adolescents using a method of contraception accounted for 2.05% (80 adolescents). Of those who were using contraception, more were using long-acting methods such as implants or norplants (1.15%, or 45 adolescents) and injections (0.70%, or 27 adolescents). To a lesser extent, some adolescents were using traditional methods of contraception (0.13%, or 5 adolescents) (see Table 3).

**TABLE 3:** Description of contraceptive methods (*N* = 3877).

Variable	*n*	%
**Use of a modern contraceptive method**		
No	3797	97.95
Yes	80	2.05
**Contraceptive method used**		
Pills	3	0.09
Intrauterine device	2	0.04
Injections	27	0.70
Male condom	3	0.07
Interrupted coitus	2	0.04
Traditional method of contraception	5	0.13
Implants or Norplant	45	1.15

### Profile of sociodemographic characteristics by modern contraceptive use

The number of adolescents using family planning is higher in rural areas than in urban areas (52 adolescents, or 1.34%). The proportion of married adolescents using contraception was 1.69%, or 66 out of 80.

Adolescents in the poor and middle wealth quintiles used the most contraceptive methods, with 28 (0.73%) and 26 (0.67%) adolescents, respectively (Table 3).

Adolescents who did not know any method of contraception accounted for 29.8%, or 1156 adolescents. Most participants reported having accessed family planning information through mass media channels such as television and radio as well as printed materials and community-based events (Table 4).

**TABLE 4:** Distribution of modern contraceptive use according to sociodemographic characteristics of Senegalese adolescents.

Variable	No (*n* = 3797)		Yes (*n* = 80)		*p* *
	*n*	%	*n*	%	
**Type of residence**	-	-	-	-	0.0214
Urban	1886	48.60	28	0.07	-
Rural	1912	49.31	52	1.34	-
**Level of education**	-	-	-	-	0.0013
No instruction	886	22.86	39	1.00	-
Primary	806	20.79	17	0.43	-
Secondary	2082	53.70	24	0.61	-
Superior	23	0.59	0	0.00	-
**Wealth quintile**	-	-	-	-	< 0.001
The poorest	580	14.97	13	0.34	-
The poorer	689	17.77	28	0.73	-
The middle	800	20.63	26	0.67	-
The richer	815	21.02	8	0.20	-
The richest	913	23.56	3	0.09	-
**Marital status**	-	-	-	-	< 0.001
Has never been in union	3103	80.03	12	0.31	-
Married	661	17.05	66	1.69	-
Cohabitation	1	0.03	0	0.00	-
Widow	1	0.03	0	0.00	-
Divorced	25	0.64	0	0.00	-
No longer living with partner/separated	6	0.16	2	0.04	-
**Religion**	-	-	-	-	0.8355
Muslim woman	3665	94.54	78	2.00	-
Christian	129	3.33	2	0.04	-
Animist	3	0.06	0	0.00	-

Note: Values are presented as *n* (%), unless otherwise specified.

* , Chi^2^ test with Rao & Scott second order correction; Wilcoxon rank sum test adapted to complex sampling designs.

Teenage girls who were unaware of any method of contraception accounted for 29.8%, or 1156 girls. The majority of respondents had obtained information about family planning from television, radio, brochures, or community meetings (Table 5).

**TABLE 5:** Modern contraceptive use by level of knowledge and source of family planning information.

Variable	No (*n* = 3797)		Yes (*n* = 80)		*p*
	*n*	%	*n*	%	
**Knowledge of a method**	-	-	-	-	< 0.001
Does not know any method	1156	29.80	0	0.00	-
Knows only traditional methods	8	0.25	0	0.00	-
Knows modern methods	2631	67.87	80	2.05	-
**Received family planning information via radio in the past few months**	-	-	-	-	< 0.001
No	3229	83.29	54	1.40	-
Yes	568	14.65	25	0.65	-
**Received information on family planning via TV during the past few months**	-	-	-	-	0.0450
No	3057	78.85	56	1.45	-
Yes	740	19.10	23	0.60	-
**Accessed information on family planning through print media over the past few months**	-	-	-	-	0.9400
No	3603	92.94	76	1.95	-
Yes	194	5.01	4	0.10	-
**Received family planning information via SMS**	-	-	-	-	0.1900
No	3641	93.91	74	1.91	-
Yes	156	4.03	6	0.14	-
**Heard about family planning through social networks (Facebook, Twitter, etc.)**	-	-	-	-	0.5600
No	3456	89.15	71	1.82	
Yes	341	8.80	9	0.23	
**Heard about family planning through brochures, posters, etc.**	-	-	-	-	0.0062
No	3425	88.36	61	1.56	-
Yes	372	9.59	19	0.48	-
**Heard about family planning at community meetings**	-	-	-	-	< 0.001
No	3336	86.06	55	1.41	-
Yes	461	11.89	25	0.63	-

TV, television.

### Factors associated with the use of contraception by adolescents

Living in a rural area was a protective factor for contraceptive use: AOR = 0.58; 95% CI: 0.34–0.96). Girls living in rural areas were less likely to use a contraceptive method than those living in urban areas. According to the results presented in Table 6, adolescents who were married had significantly higher odds of using modern contraceptive methods compared to their unmarried peers (AOR = 20.61; 95% CI: 11.43–37.14). Similarly, girls who were no longer living with their partners showed an even stronger association with contraceptive use (AOR = 105; 95% CI: 23.6–467.21). In addition, adolescents belonging to the lower economic quintile were more than twice as likely to adopt modern contraception (AOR = 2.35; 95% CI: 1.35–4.08). The likelihood of contraceptive use was also higher among Christian adolescents compared to Muslim girls (AOR = 3.41; 95% CI: 2.52–18.19).

**TABLE 6:** Determinants of contraceptive method use.

Characteristics	AOR†	95% CI	*p*
**Age (years)**	1.00	-	-
**Place of residence**			
Urban	1.00	-	-
Rural	0.58	0.34–0.96	0.037*
**Level of education**			
No instruction	1.00	-	-
Primary	0.94	0.54–1.63	0.830
Secondary	1.16	0.67–1.99	0.580
**Wealth quintile**			
The poorest	1.00	-	-
The poorer	2.35	1.35–4.08	0.002*
The middle	1.63	0.84–3.18	0.140
The richer	1.10	0.47–2.58	0.810
The richest	0.68	0.19–1.96	0.410
**Marital status**			
Has never been in union	1.00	-	-
Married	20.61	11.43–37.14	< 0.001*
No longer living with partner or separated	105.00	23.6–467.21	< 0.001*
**Religion**			
Muslim woman	1.00	-	-
Christian	3.41	2.52–18.19	< 0.001*

AOR, adjusted odds ratio; CI, confidence interval.

* , *p* < 0.05 statistically significant; **, *p* < 0.01; ***, *p* < 0.001.

† , Reference category.

## Discussion

The findings of this study underscore a significant gap between the sexual activity of adolescent girls in Senegal and their use of modern contraceptive methods. Although nearly one in five adolescents reported having initiated sexual intercourse – with some as young as age 9 years – current utilisation of modern contraceptive methods was reported by only 2.05% of the adolescents. This discrepancy reflects a broader trend in sub-Saharan Africa, where a growing number of adolescents, particularly from disadvantaged backgrounds, become sexually active at an early age, often without access to adequate reproductive health services.^14^

Compared to other African countries, the level of contraceptive use among Senegalese adolescents remains markedly low. While prevalence among adolescents varies greatly across the continent – from 4% in Chad to over 60% in Namibia – the majority of countries still report rates below 50% among sexually active adolescents.^15^ This indicates persistent structural and social barriers that hinder adolescent access to contraception despite global and national commitments to reproductive health rights.

Notably, in this study, long-acting methods such as implants and injectables were more commonly used among adolescents than oral contraceptives, a pattern that differs from trends in many Western countries where pills remain the preferred method.^16^ This divergence may reflect differences in access, provider preferences, or programmatic emphasis in method provision.

The role of knowledge and perceptions also merits attention. While most respondents reported exposure to family planning messages via mass media and community-based platforms, previous studies have shown that such knowledge alone does not necessarily translate into use.^17^ Access to reliable and youth-adapted information, including discussions about contraceptive options and their side effects, is essential. In Senegal, efforts have been made to include adolescents in national communication strategies, notably through the establishment of a dedicated reproductive health division. However, bridging the gap between awareness and practice remains a challenge.^18,19^

Several factors were found to be associated with contraceptive use. Adolescents residing in rural areas were significantly less likely to use modern contraceptives (AOR = 0.58; 95% CI: 0.34–0.96]). This finding aligns with evidence from other contexts showing greater barriers to access in rural compared to urban settings, including geographic distance, limited availability of youth-friendly services, and provider bias.^20^ Interestingly, adolescents in the lowest wealth quintile were more likely to use contraception (AOR = 2.35; 95% CI: 1.35–4.08), a finding that contrasts with previous studies in older populations where higher socioeconomic status is often linked to increased use.^21^ This may suggest successful targeting of the poorest by public health programmes, but it also raises concerns about potential disparities in method quality and informed choice.

Marital status also played a key role. Married adolescents and those who were separated from their partners were significantly more likely to use modern contraceptives (AOR = 20.61; 95% CI: 11.43–37.14 and AOR = 105; 95% CI: 23.6–467.21, respectively). Similar trends have been observed in other studies, which attribute this to stronger social networks and community structures that facilitate access to services among married adolescents.^22,23^

Religious affiliation appeared as another important factor. Christian adolescents were more likely to use contraception than their Muslim peers (AOR = 3.41; 95% CI: 2.52–18.19). While religion itself may not directly oppose family planning, the interpretation of religious norms and their perceived compatibility with contraceptive use often shape individual behaviour.^24,25^ In this context, engaging religious leaders and integrating culturally sensitive messaging into health promotion efforts may help reduce resistance and misperceptions.

In sum, these findings highlight the multifactorial nature of contraceptive use among adolescent girls. Addressing the unmet need for contraception in this population requires tailored interventions that are sensitive to social, economic, cultural, and geographic determinants. Programmes should not only ensure the availability of contraceptive methods but also create enabling environments where adolescents can make informed and autonomous decisions about their reproductive health.

### Strengths and limitations

One notable strength of this study is the use of data from the Senegal DHS, which provides nationally representative estimates. This enhances the external validity of the findings, allowing for generalisation to the broader population of adolescent girls of reproductive age in Senegal. Nonetheless, the study is subject to certain limitations.

Given that the analyses were based on cross-sectional data, the findings reflect associations rather than causal relationships. To deepen the understanding of factors influencing adolescents’ use of modern contraception, a complementary qualitative study could help capture the social dynamics and contextual realities that shape their behaviours.

## Conclusion

This study provides critical insights into the persistently low use of modern contraceptive methods among adolescent girls in Senegal, despite early sexual debut and relatively widespread exposure to family planning information. The findings emphasise the need for a nuanced and multidimensional approach to adolescent reproductive health, one that goes beyond awareness campaigns to address deeper sociocultural, economic and systemic barriers. Tailored interventions should prioritise equitable access to youth-friendly contraceptive services, particularly for unmarried adolescents, those living in rural areas, and those from disadvantaged backgrounds. Efforts to engage communities – including parents, partners and religious leaders – are essential to fostering an environment that supports adolescent autonomy and informed decision-making.

By centring adolescent girls in the design and delivery of reproductive health programmes, Senegal can accelerate progress towards its national and international commitments on sexual and reproductive health while also advancing gender equity and health outcomes for future generations. In addition to programmatic responses, future research should incorporate qualitative socio-anthropological approaches to explore and better understand the sociocultural norms, perceptions and community-level dynamics that hinder contraceptive uptake among adolescents. Such perspectives are essential to designing interventions that are not only evidence-based but also socially acceptable and culturally embedded.
